# Viscoelastic response of confined powder under large strain oscillations, characterized by its noise temperature

**DOI:** 10.1140/epje/s10189-023-00310-w

**Published:** 2023-07-15

**Authors:** Rishab Handa, Christian Wagner, Jorge Eduardo Fiscina

**Affiliations:** 1 Powderreg Project Cross-Border Cooperation, European Union, 54505 Vandoeuvre-lès-Nancy, France; 2 Experimental Physics, Saarland University, Im Stadtwald, 66123 Saarbrücken, Saarland Germany; 3 Department of Physics and Materials Science, University of Luxembourg, L1511 Luxembourg, Luxembourg

## Abstract

**Abstract:**

We report a study on granular matter with and without small additions of silicon oil, under low-frequency and large amplitude oscillatory shear strain under constant normal pressure, by running experiments with a rotational rheometer with a cup-and-plate geometry. We analysed the expansion with the Chebyshev polynomials of the orthogonal decomposition of stress–strain Lissajous–Bowditch loops. We found the onset of the strain amplitude for the yielding regime indicated a regime change from filament-like structures of grains to grain rearrangements for the dry granulate and from oscillations to the breaking and regeneration of liquid bridges for wet granulates. We have shown that this viscoelastic dynamics can be characterized by a noise temperature following Sollich et al. (Phys Rev Lett https://doi.org/10.1103/PhysRevLett.78.2020, 1997). The analysis of the first harmonics of the Chebyshev expansion showed that the state of disorder of dry and wet granular matter in pre-yielding and yielding regimes involved ensembles of different inherent states; thus, each of them was governed by a different noise temperature. The higher-order harmonics of the Chebyshev expansion revealed a proportionality between the viscous nonlinearity and the variation in the elastic nonlinearity induced by the deformation, which shows the coupling between the elastic deformation and the viscous flow of mesoscopic-scale structures.

**Graphic abstract:**

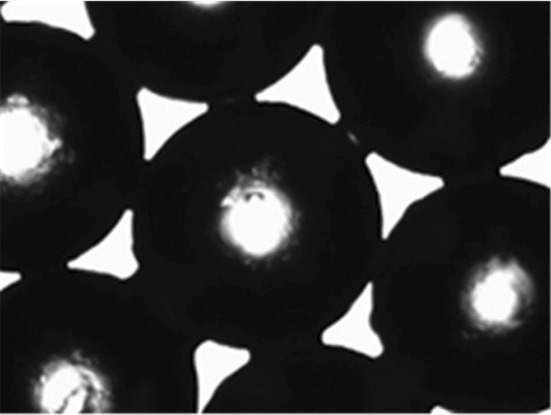

## Introduction

Plasticity models based on the Coulomb friction approach have been proposed to study different powder flow regimens [1]. The constitutive equations for describing how the granulate flows and deforms were derived from this picture using the Mohr–Coulomb extended theory; this is achieved through a plastic potential and by including the dilatancy and consolidation laws of the granular media [2]. The flowing powder is approached as a Mohr–Coulomb or frictional fluid [3–8]. Given a flowing ensemble of rigid beads of density $\rho$ and diameter *d*, under confinement pressure *P*, the dimensional analysis reveals a selection rule between the shear stress $\tau$ and the shear rate $\dot{\gamma }$: $\tau = \mu (I)\cdot P$, where $\mu$ is an effective friction coefficient dependent on a dimensionless number *I* [9, 10]. To complete this model, the packing fraction is set by a dilatancy law $\phi =\phi (I)$. The inertial number *I* is given by the ratio of two time scales $I=\dot{\gamma }\cdot D/\sqrt{P/\rho }$, the inertial time scale and the time scale for rearrangements. Further, this number permits the attainment of the evaluation of an apparent viscosity $\eta _{app}=\mu (I)\cdot P/\dot{\gamma }$. The experimental conditions drive to a steady homogeneous shear state, which enables the measurement of macroscopic constitutive laws; it is then possible to write the dilatancy and consolidation laws in terms of the inertial number [9].

These studies represent an important advancement in describing different flow configurations under a controlled shear rate. The validity of this frictional picture, also known as $\mu (I)$ rheology, is further extended for cohesive granular materials under flow [11]. Despite the effectiveness of this model in describing frictional fluids, it was found to be an improper description of the non-locality of the powder rheology. Subsequently, a granular fluidity model emerged, where the fluidity is the inverse of viscosity, defined as $g=\dot{\gamma }/\mu (I)$. The fluidity *g* is only a mathematical artefact without a real physical meaning; since it considers the non-locality of rheological events, it worked to model many flow scenarios where $\mu (I)$ rheology does not work properly [12–14].

Boutreux and De Gennes [15] developed a free volume model and found that the relation between the compaction and the grain mobility can be described by the Vogel–Fulcher–Tammann equation, while Lumay and Vandewalle [16] reported the experimental evidence. The important experiments conducted by D‘Anna revealed the similarity between the glassy state and jammed granular matter, since it was found that the route to the jammed transition follows a modified Vogel–Fulcher–Tamman behaviour [17, 18]. Philippe and Bideau [19] concluded that in the case of a weak excited granulate, its compaction and the relaxation of an out-of-equilibrium thermal system exhibit similar behaviour. The compaction of the excited granular bed can be fitted by a stretched exponential function or the Kohlrausch–Williams–Watts law (KWW), where the relaxation time depends on the time scale for the rearrangement. The resistance to flow occurs when the bulk granulate goes from one configuration to the next. This rearrangement process under gravity drives the grains to be jammed in a new configuration; thus, the compaction dynamics of dense granulates is related to the energy that is necessary to dissipate to go from one configuration to the other. Lu et al. [20] contributed an important step in this research line by stating the relationship between the steady-state rheology and the compaction behaviour of powders as part of a more general jamming theory. Their model is based on granular compressibility, retaining the Coulomb yield conditions and dilatancy behaviour. A first view of compaction and rheological tests suggests that the time scale $\tau _{r}$ for the rearrangement is to be related with the jumping energy for a single void. From shear flow experiments, they deduced the non-equilibrium equation of state 1, relating the confinement pressure *P* with the volume fraction written as the flowing shear band volume referenced to the dynamic random close packing volume $\epsilon =V-V_{\text {RCP}}$ and then normalized to the minimum free volume $\epsilon _{0}$, with $\kappa =\left[ \partial V/\partial P \right] _{\dot{\gamma }}/V$ being the compressibility, and *C* and $\dot{\gamma _{0}}$ the constants related to the dependence of the volume fraction to the shear rate, dependent on the confinement pressure and rearrangement events [20, 21].1The confinement pressure in the equation of state 1 could be read as an energy density, in which the inverse of the compressibility, in a mean-field picture, is related to the viscoelastic energy landscape of the granular assembly.

Following these findings, we found it interesting that an alternative approach for understanding powder flow has been shown to experimentally complement the research of emerging concepts in non-equilibrium thermodynamics. A rheological model for soft glassy materials (SGM) was proposed by Sollich et al. [22] and developed by Fielding [23, 24], in which the model describes them as an ensemble of elastic elements, each storing elastic energy, whose dynamics is set by a parameter called the noise temperature. The elastic noise of these elements was depicted in the model as the jumping over strain-modulated energy barriers. The noise temperature governing their dynamics can be understood as a genuine thermodynamic temperature $E_{0}=k_{B}\cdot \Theta$ [25], as in the frame of the shear transformation zone (STZ) theory, developed by Falk [26]. During shear deformation of dry granulates, mesoscopic-scale rearrangements can be identified as slow configurational degrees of freedom or ‘inherent states’ far from equilibrium with a configurational non-thermal kind of thermostat. Those degrees of freedom maximize a configurational entropy; thus, their viscoelastic state of disorder should be characterized by a configurational temperature. The two aforesaid approaches were presumed distinct and unrelated; however, the SGM rheology can bring physical understanding from first principles to the $\mu$-rheology originated in fluid mechanics to predict the physical parameters that influence the flow, with a profound orientation towards its practical and industrial applications.

**Fig. 1:**
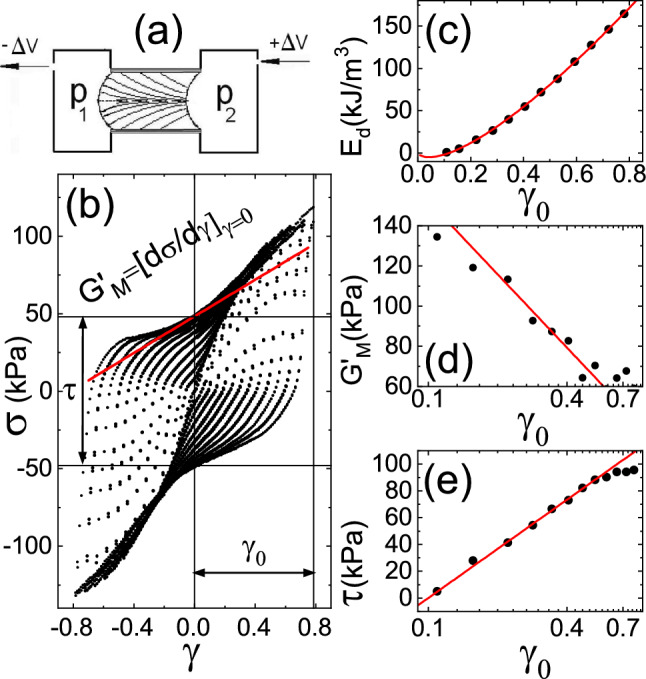
Oscillatory rheology on monodisperse dry sand of $d=(145 \pm 5)\,\mu {\text {m}}$ carried out by pushing sand through a tube experiment as it was reported in [27]: **a** schematic representation of the cylindrical test cell containing the sample and two adjacent chambers filled with water where the pressures $p_{1}$ and $p_{2}$ are measured. **b** differential pressure $\sigma = p_{1}-p_{2}$ versus strain $\gamma$. A family of Lissajous loops from a range of strain amplitudes between $\gamma _{0}=0.1$ and $\gamma _{0}=0.8$. The parameters of the last loop are indicated as follows: the loop’s amplitude $\tau$, the tangent modulus $G'_{\text {M}}$ and the strain amplitude $\gamma _{0}$. **c** Dissipated energy $E_{d}= \oint \sigma \,d\gamma$, **d** tangent modulus $G'_{M}$, and **e** amplitude of the loop $\tau$ vs. strain amplitude $\gamma _{0}$

Contributing to this research line, we carried out our experimental studies in particular, the noise temperature [27] and the connection between compaction experiments and rheology [28, 29]. Our previous studies on oscillatory rheology on wet and dry granulates were carried out with a so-called tube rheometer [27], a device described first in the thesis by Geromichalos [30]. The device we used is schematically depicted in Fig. 1a. The granulate is poured in to a cylindrical test cell of diameter *D* and equal longitude $D=L$, sealed with latex membranes of ${300}\,\mu {\text {m}}$. Two adjacent chambers are filled with water and two syringe-pistons drive the membranes quasi-statically in an oscillatory manner between volumes $\pm \Delta V$, while keeping the global packing fraction $\phi$ of the granulate constant. Pressure sensors inside the chambers measure the pressures $p_{1}$ and $p_{2}$ complete the set-up. The granulate is then pushed, enforcing a Poiseuille profile; thus, the maximum amplitude can be estimated as $\gamma _{0} = 32 \Delta V/ \pi D^3$ [31]. Running this experiment for a range of $\gamma _{0}$ permitted us to characterize the granulate from a family of Lissajous loops $\sigma -\gamma$, where $\sigma = p_{1}-p_{2}$, as shown in Fig. 1b, and in this particular case study, for a sample of monodisperse dry sand of diameters in the range of $d=(145 \pm 5)\,\mu {\text {m}}$ and with global packing fraction $\phi =0.63$. Nonlinear loops occur for $\gamma _{0}>0.1$, which corresponds with nonlinear events in the case of the dry granulate related to rearrangements in the mesoscopic scale of force chains, which occurs for displacements of roughly larger than one grain diameter. In Fig. 1b, the important parameters of the loop are indicated, with its amplitude $\tau$, the strain amplitude $\gamma _{0}$ and the minimum strain elastic or tangent modulus at $\gamma =0$, $G'_{\text {M}}={\text {d}}\sigma /{\text {d}}\gamma |_{0}$, both related to the storage elastic energy and the area of the loop $E_{\text {d}}=\oint \sigma \,{\text {d}}\gamma$, the dissipated energy per circle. In Fig. 1c, d and e, our most important finding is shown, which is the representation of $E_{\text {d}}$, $G_{\text {M}}$ and $\tau$ in function of the strain amplitude $\gamma _{0}$, in the nonlinear rage from $\gamma _{0}^\text {onset}$. The corresponding fittings with Eq. 2 revealed that all of them are related to a unique parameter, identified as the noise temperature $E_{0}$, where $G_{0}$ is a constant.2For this particular case, the fit parameters resulted in $\tau _{0}=(53 \pm 2)\,\hbox {kJ}/\hbox {m}^{3}$ and $\gamma _{0}^{\text {onset}}=(0.12 \pm 0.01)$; being $\tau _{0}\equiv G_{\text {M}}^{0} \equiv E_{0}$, as the fittings validated in Fig. 1.

We also found in our previous work [27], for the wet granulate, the noise temperature is in good agreement with the estimation of the energy loss occurring at the mesoscopic scale when the liquid bridge breaks and regenerates; whereas, for dry powders, these mesoscopic oscillators are the force chains that lose energy when branching out.

At Liege University, in collaboration with Lumay and Vandewalle [28, 29], we conducted compaction experiments using the same monodisperse sand as in [27] with small amounts of different fluids. For these experiments, we used an energetic approach by assuming the existence of an energy barrier *B*. The model considers the mechanical energy per grain $\Xi$ injected in the granular bed at each tap, being the characteristic relaxation time measured by $\tau _{1/2}$, i.e. the time required to reach half of the difference between the initial and asymptotic packing fraction values. This resulted in Eq. 3:

3 $$\begin{aligned} {\begin{aligned} \tau _{1/2}=\frac{1}{\alpha }\cdot \exp \bigg (-\frac{\Xi }{B}\bigg )\cdot \bigg [E_{i}(\frac{2B}{\Xi })-E_{i}(\frac{B}{\Xi })\bigg ] \end{aligned}} \end{aligned}$$

where $\alpha$ is a dissipation factor and $E_{i}(y)= \int _{-\infty }^y f(\theta ){\text {d}}\theta$ is the exponential integral of the dimensionless variable *y*. For granulates with small additions of fluids of surface tension $\gamma _{\text {f}}$, we found $B/\Xi =\gamma _{\text {f}}/\gamma _{f}^{0}$; where $\gamma _{\text {f}}^{0}$ is a constant. The same result was found from the experiments with the tube rheometer, where the noise temperature can be tuned with the surface tension $B\equiv E_{0}\propto \gamma _{\text {f}}$. This observation highlights the relation of the noise temperature that governs the rearrangement between jammed states. This was remarked by Lu et al. [20] as a key to understand jamming within the relation between rheological tests and compaction experiments.

We continued these investigations, and we report here oscillatory rheology studies with a rotational rheometer, implementing a large amplitude oscillatory shear strain (LAOS-strain). This allowed us to explore powder flow nonlinearities from the non-sinusoidal response we derived by a sinusoidal excitation. This protocol first used by Gemant et al. [32] is clearly discussed by Rogers [33] as ‘hard to interpret’ despite the large body of literature related to polymer research that attempts to explain the viscoelasticity of polymers; for example, one study relates their macromolecular structure and polymer branching to the higher harmonics evaluated from the nonlinear response [34]. Measurements are represented as a family of Lissajous–Bowditch (LB) loops in the planes $\sigma -\gamma$ and $\sigma -\dot{\gamma }$ obtained from orthogonal stress decomposition [35] and expanded using Chebyshev functions [36], from which the Chebyshev coefficients are analysed and interpreted [37–40].

There are concerns about the physical explanation of nonlinear responses in relation to this methodology [41–44]; however, in this study, we report the relation of the Chebyshev coefficients with the energy landscape of the granular assembly as it emerges from the experimental results. In this report, we use the knowledge from our previous work with the ‘tube rheometer’; powder compaction experiments of dry and wet granular assemblies, where we found that the onset of the yielding range $\gamma _{0}^\text {onset}$ occurred for a deformation provoking a displacement of the order of the diameter of one grain, followed by rearrangements of grains happening mostly in cages of a size of four grain diameters; and the breaking and regenerating of liquid bridges in the case study of the wet granulates [27–29]. In our previous analysis of the LAOS-strain measurements obtained with the ‘tube rheometer’, we were interested in parameters that characterize the granulate in average. Our findings were reproducible within a wide range of strain rates, from quasi-static regime, where we applied step-by-step deformation smaller than the diameter of one grain, to continuous deformation, where we tested shear rates until ${0.1}\,s^{-1}$, establishing a steady state without observing differences in the LB-loop, i.e. in the loop area or dissipating energy. In this study, we did similar oscillatory rheology experiments with a rotational rheometer under constant normal force, without deforming the grains and enough to guarantee the contact between the sensor and the granulate. From the results of these experiments, we examine the Chebyshev coefficients in relation to the SGM model, referred to in previous paragraphs, applied to powders under confinement, characterizing them with the noise temperature, without interpreting them in the usual way, i.e. as directly related to transient changes affecting the intracycle strain distribution.

This report is organized as follows: Sect. 2 describes the granular media preparation and the experimental procedures, while in Sect. 2.1, we first briefly overview Fourier transform rheology used to evaluate our $\sigma -\gamma$ Lissajous–Bowditch loop measurements. In Sect. 2.2, we present the granular assemblies used in the experiments. In Sect. 2.3, we present a description of the set-up and experimental protocol, based on a rotational rheometer for measuring Lissajous loops of the sample under confinement, similar to the set-up used by Lu et al. [21]. Given the set-up of the cup-and-plate geometry, we estimated a correction for the rheometer calibration. In Sect. 2.4, we provide, for the case of wet granulates, an estimation of the rupture energy of capillary bridges. Section 3 corresponds to the report of the experimental results and their expansion with Chebyshev polynomials, where in 3.1, we proposed an equation of state based on the first harmonic elastic and viscous moduli, and in 3.2 we analysed the contribution of the higher-order nonlinearities. In Sect. 4, we offer stand-alone conclusions on the reported results and an outlook considering our previous contributions to this research line in the framework of the SGM rheology.

## Materials and methods

### **Fourier transform rheology**

**Fig. 2:**
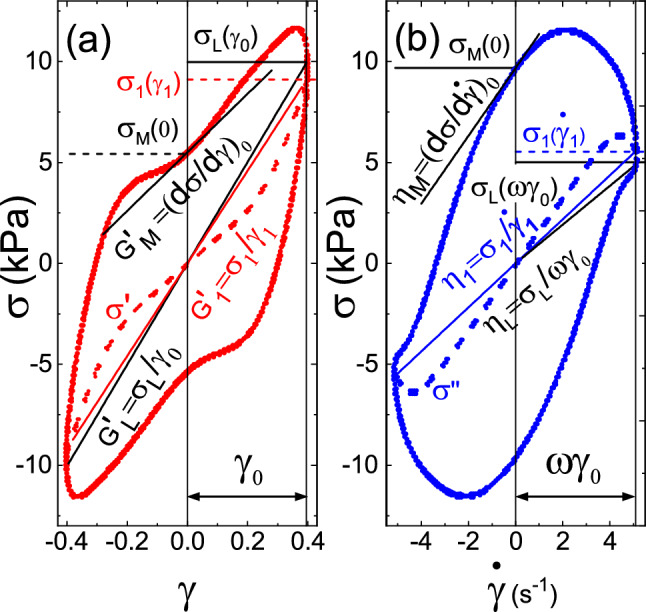
Orthogonal decomposition of a Lissajous–Bowditch loop from a LAOS-strain experiment for polystyrene beads of ${500}\,\mu {\text {m}}$ diameter with small additions of silicon oil, following Cho et al. [35]: **a** stress versus strain and elastic stress $\sigma '$ and **b** stress versus strain rate and viscous stress $\sigma ''$ vs strain rate. These loops permit us to show the nonlinear moduli as the derivatives, and the slopes at the coordinates indicated in (**a**) and (**b**) (see text for detailed explanation)

A rotational rheometer was used in large amplitude oscillatory shear strain mode LAOS-strain. The stress response was measured for an input signal of a temporal sinusoidal strain, given by $\gamma (t) = \gamma _0\cdot \sin {(\omega t)}$, with $\omega$ the imposed oscillation frequency, *t* time, and $\gamma _{0}$ the strain amplitude [37–40]. Being $\dot{\gamma }(t) = \gamma _0\cdot \omega \cdot \cos {(\omega t)}$ the evaluated strain rate, the elastic and viscous moduli, $G^{'}$($\omega$) and $G^{''}$($\omega$) can be determined. A typical representation of a LAOS test is the Lissajous–Bowditch plots, where the cyclic variations of shear stress as a function of strain (elasticity) and the evaluated strain rate (viscosity). These are displayed in Fig. 2 [38]. In Fourier transform (FT) rheology, not only the first harmonics but also higher harmonics (odd only) contain valuable nonlinear information [37, 38]. Only odd harmonics are considered because of the assumption that stress–strain exhibit odd symmetry with respect to the propagation of shear strain or rate [38]. Also, the presence of even harmonics in the output response would imply that the deforming material sticks to the boundary wall [45]. We introduce the working of FT-rheology and expansion with Chebyshev polynomials of the stress–strain Lissajous–Bowditch (LB) loops. Following the article by Wilhelm et al. [46], the Fourier series expansion of the stress is given in elastic scaling in Eq. 4:

4 $$\begin{aligned} \sigma (t; \gamma _{0}, \omega ) = \sum _{n, \,\text {odd}} \left[ a_{n}\cdot \cos (\omega _{n} t)+b_{n}\cdot \sin \left( \omega _{n} t\right) \right] \nonumber \\ \end{aligned}$$

where $\omega$ and $\gamma _{0}$ are the independent variables, $\omega _n=2 \pi n$ is the angular frequency, $a_{n}$ and $b_{n}$ are the Fourier coefficients of the $n{\text {th}}$ harmonic as they are defined in Eq. 5, where it is also shown their relation with the corresponding viscoelastic moduli:

5 $$\begin{aligned} {\begin{aligned} a_{n}&=\frac{2}{T}\int _{0}^{T} \sigma (t)\cdot \cos (\omega _{n} t) {\text {d}}t = \gamma _{0}\cdot G_{n}^{''} \\ b_{n}&= \frac{2}{T}\int _{0}^{T} \sigma (t)\cdot \sin (\omega _{n} t) {\text {d}}t = \gamma _{0}\cdot G_{n}^{'} \end{aligned}} \end{aligned}$$

Furthermore, the intensity of the $n{\text {th}}$ harmonic is defined in 6 as:

6 $$\begin{aligned} {I_n=\sqrt{a _ {n}^2 + b _ {n}^2}} \end{aligned}$$

To characterize the amount of nonlinearity, the relative intensity of the higher odd harmonics compared with the first harmonic is commonly used [47], as shown in 7:

7 $$\begin{aligned} I_{n1} = \sqrt{\frac{a _ {n}^2 + b _ {n}^2}{a _ {1}^2 + b _ {1}^2}} \end{aligned}$$

In a standard rheological test, only $G_{1}'$ and $G_{1}''$ are measured and they fully describe the linear viscoelastic response of the material. Nonlinear properties can be determined by FT-rheology, but an open question is the physical interpretation of the higher-order harmonics [38]. Furthermore, fitting a Fourier response, for example $A=a_{0}\cdot f_{0}(x)+a_{1}\cdot f_{1}+a_{2}\cdot f_{2}+\cdots$ without violating the limit of orthogonality over the finite integration domain $[-1,+1]$ results in a half sided Fourier transform, which is similar to a complex Laplace transformation. Hence, one loses half of the measured information [46]. From the work by Cho et al. [35] on orthogonal stress decomposition and further extension by Ewoldt et al. [36], the relation between elastic $\sigma ^{'}$ and viscous stresses $\sigma ^{''}$ and the Fourier decomposition can be written as in Eq. 8:

8 $$\begin{aligned} \begin{aligned} \sigma '&\equiv \frac{\sigma (\gamma , \dot{\gamma })-\sigma (-\gamma , \dot{\gamma })}{2} \\ {}&=\gamma _{0}\sum _{n, \,\text {odd}} G_{n}^{'}(\omega , \gamma _{0})\, \cdot \text {sin} (n\omega t)\ \\ \sigma ''&\equiv \frac{\sigma (\gamma , \dot{\gamma })-\sigma (\gamma , -\dot{\gamma })}{2} \\ {}&=\gamma _{0}\sum _{n, \,\text {odd}} G_{n}^{''}(\omega , \gamma _{0})\cdot \text {cos}(n\omega t) \end{aligned} \end{aligned}$$

where $G_{n}^{'}$ and $G_{n}^{''}$ are the amplitudes of the higher-order viscoelastic moduli. The total oscillatory shear stress is then given by $\sigma (t) = \sigma ^{'} (t) + \sigma ^{''} (t)$, which leads to the alternative derivation of stress decomposition compared to the classical FT-Rheology [39]. McKinley and Ewoldt identified the Chebyshev polynomials, a cogent choice for the decomposition as they exhibit stress–strain symmetry at vanishing strain amplitudes, they are orthogonal over a finite domain $[-1,+1]$ and they can be thoroughly correlated to the Fourier coefficients. Since the Chebyshev polynomials have the advantage of being orthogonal to all the modes of odd harmonic signals, this allows us to efficiently describe the nonlinear viscoelastic moduli in a series of basis functions, thereby nullifying the interference of one mode with another and offering a near-optimal polynomial interpolation of higher harmonics [36].

The elastic and viscous contribution to the measured stress is given in the Chebyshev basis as follows in Eq. 9:

9 $$\begin{aligned} {\begin{aligned} \sigma '(x)&= \gamma _{0}\sum _{n: \,\text {odd}} e_{n}(\omega , \gamma _{0}) T_{n}(x) \ \\ \sigma ''(y)&=\omega \cdot \gamma _{0}\sum _{n: \,\text {odd}} v_{n}(\omega , \gamma _0) T_{n}(y) \end{aligned}} \end{aligned}$$

where $x=\gamma /\gamma _{0}$, $y=\dot{\gamma }/(\omega \cdot \gamma _{0})$ and $T_{n}(x)$ is the $n^{\text {th}}$-order Chebyshev polynomial of the first kind, $e_{n}(\omega , \gamma _{0})$ and $v_{n}(\omega ,\gamma _{0})$ are the elastic and viscous Chebyshev coefficients, respectively. This particular Chebyshev basis-set permitted Ewoldt et al. [36] to evaluate the coefficients $e_{n}$ and $v_{n}$ in the strain or strain rate domain by the following relations with the Fourier coefficients in the time domain, Eq. 10:

10 $$\begin{aligned} {\begin{aligned} e_{n} = G_{n}^{'}(-1)^{(n-1)/2}, \,\ \omega \cdot v_{n} =G_{n}^{''}, \quad n: \,\text {odd} \, \end{aligned}} \end{aligned}$$

An example for an LB-loop orthogonal decomposition using aforementioned procedure is shown in Fig. 2 [35], including the definitions for viscous and elastic moduli, a geometrical extraction of nonlinear elastic moduli and dynamic viscosities via tangential slopes are shown [36, 38]. They are rigorously defined by Eqs. 11 to 14:

11 $$\begin{aligned} {\begin{aligned} G_{\text {M}}'\equiv \Bigl [\frac{d\sigma }{d\gamma }\Bigr ]_{\begin{array}{c} \gamma =0 \end{array}}&=\sum _{n: \,\text {odd}} nG_{n}^{'} \\ {}&=e_1-3e_3+\cdots \end{aligned}} \end{aligned}$$

12 $$\begin{aligned} {\begin{aligned} G_{\text {L}}^{'}\equiv \Bigl [\frac{\sigma }{\gamma }\Bigr ]_{\begin{array}{c} \gamma =\pm \gamma _{0} \end{array}}&= \sum _{n: \,\text {odd}} G_{n}^{'}(-1)^{\frac{(n-1)}{2}} \\ {}&=e_1+ e_3+\cdots \end{aligned}} \end{aligned}$$

13 $$\begin{aligned} G_{\text {M}}^{''} \equiv \omega \cdot \eta _{\text {M}}^{'}= & {} \omega \cdot \Bigl [\frac{d\sigma }{d\dot{\gamma }} \Bigr ]_{\begin{array}{c} \dot{\gamma }=0 \end{array}} =\sum _{n: \, \text {odd}} n\cdot G_{n}^{''}(-1)^{\frac{(n-1)}{2}}\nonumber \\ {}= & {} \omega \cdot v_1-3\omega \cdot v_3+\cdots \end{aligned}$$

14 $$\begin{aligned} \begin{aligned} G_{\text {L}}^{''} \equiv \omega \cdot \eta _{L}^{'}&=\omega \cdot \Bigl [\frac{\sigma }{\dot{\gamma }}\Bigr ]_{\begin{array}{c} \dot{\gamma }=\pm {\gamma }_{0}\omega \end{array}} \\&=\sum _{n: \,\text {odd}} G_{n}^{''}=\omega \cdot v_1+\omega \cdot v_3+\cdots \end{aligned}\nonumber \\ \end{aligned}$$

where $G_{M}^{'}$ is the minimum strain elastic or tangent modulus at $\gamma = 0$. Referring to the tangent at the coordinate $\sigma _{M}(0)$, $G_{L}^{'}$ is the large strain elastic or secant modulus at $\gamma =\gamma _{0}$ (secant at $\sigma _{L}(\gamma _{0})$) (Fig. 2a). Similarly, $\eta _{\text {M}}^{'}$ is the minimum-rate viscosity, the tangent at $\sigma _{M}(\dot{\gamma }=0)$, or the tangent loss modulus $G_{\text {M}}^{''}$ and $\eta _{L}^{'}$ is the large-rate dynamic viscosity, the secant at $\sigma _{L}(\omega \gamma _{0})$, or the secant loss modulus $G_{\text {L}}^{''}$ (Fig. 2b). $G_{1}^{'}$ and $\eta _{1}^{'}$ are the first harmonic elastic modulus and dynamic viscosity, respectively, evaluated at the coordinates of the intersection of the loops with the symmetry lines, corresponding to the elastic and viscous stress, in Fig. 2a $\sigma _{1}(\gamma _{1})$ and (b) $\sigma _{1}(\dot{\gamma _{1}})$.

As Rogers discussed in his work [43], a complete interpretation of the Chebyshev coefficients remains unpublished, as the research on this methodology progressed there was a tendency to carry out interpretations of intracycle and dynamical changes based on relations between the Chebyshev coefficients, which gave rise to confusion in some case studies, as so happened in the case of the strain softening/strain hardening paradox resolved by Mermet-Guyennet et. al. [48]. They first applied this way to analyse LAOS experiments, called ‘sequence of physical processes’ (SPP), to yield stress fluids [41], also developing solid arguments from the discussion of nonlinear theoretical models [42], where it was shown that the symmetry assumptions for the Chebyshev functions are too restrictive in relation to such models, this is not making invalid to analyse LAOS measurements applying the stress decomposition plus the expansion with Chebyshev functions, but again it is signalling that despite of the Chebyshev coefficients are hard to interpret, their interpretation must be the proper one. The SPP methodology has been shown to provide great clarity in understanding the intracycle structural and dynamical changes as they happen in granular materials as local rearrangements, breakage of liquid bridges and reformation. This provides detailed microstructural interpretations with the time-dependent rheological behaviour of the material throughout the oscillation cycle; however, in this study, we are only interested in the characterization of the energy landscape of the confined granulate, where it seems, as it emerged from our analysis of the experiments reported here, that the Chebyshev coefficients as an ensemble of viscoelastic moduli are related to average characteristic energy densities describing the energy landscape of the granulate. Analysis using the sequence of the physical processes in relation with soft glassy rheology, as it was applied by Park and Rogers [44], will be reported in future manuscripts related to our research of the granular system presented here.

### **Materials**

In the reported experiments, we used polystyrene beads of ${500}\,\mu {\text {m}}$ diameter, purchased from Microbeads, USA (trade name: Dynoseeds®). The fluid content of the mixture is defined as the ratio between the liquid volume and the volume occupied by the beads. The silicon oil Shin Etsu SE KF-6011 was dispersed in the volume of beads to get a paste with ${2}{\text { vol}\%}$ of fluid. The viscosity of the pure oil is $\mu =0.18\,\hbox {Pa s},$, its surface tension$\gamma _{oil}={21}\,\hbox {mN}\,{\hbox {m}^{1}}$ at ${21}{}^circ \hbox {C}$, and its density $\rho ={1070}\,\hbox {kg}\,\hbox {m}^{-3}$, very close to the density of the Dynoseeds’ beads. As we discussed in our previous work [27] explained in the introduction Sect. 1, i.e. for the wet sand, its characteristic energy or noise temperature stays constant in the range of existence of the liquid bridge network, in which a water content of $0.01\lesssim \ w \lesssim 0.03$ results in good agreement with the findings of Scheel et al [49]. Inspired in this, we explore also a similar range of oil content in which we got a stable liquid network; thus, we decided to prepare the wet granulate by mixing the beads with ${2}\,{\text {vol}\%}$, by stirring the mixture until noticing all the beads covered with a layer of silicon oil enough to get into the pendular state, being sure of the homogeneity of the liquid bridge network and the very well-defined energy landscape.

**Fig. 3:**
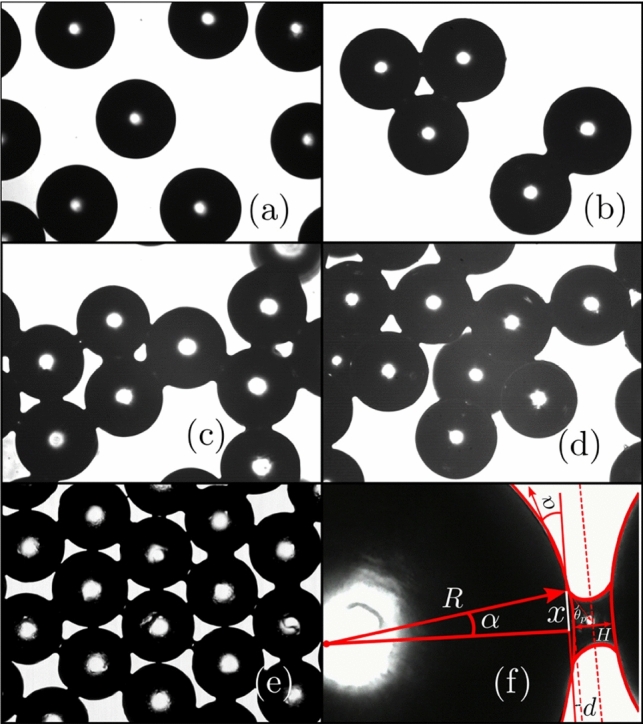
Polystyrene beads of diameter ${500}\,\mu {\text {m}}$, two dimensional layer. **a** Dry and **b** wet beads form dimers and trimers. **c** beads wetted with ${2}\,{\text {vol}\%}$ of silicon oil in relation to the volume of the beads and **d** with ${3}{\text { vol.}\%}$. **e** s monolayer of wet beads with coordination 6. **f** geometrical representation of the capillary bridge between two beads. Images were inspired by Kudrolli [50]

Figure 3 shows the micrographic bright-field images of the polystyrene granulate used in this study. Figure 3c, d shows partially saturated polystyrene beads. A small fraction of interstitial fluid is sufficient to form a capillary pendular bridge causing a spring-like action and cohesivity between grains [51]. In this so-called pendular regime, the shear stress largely depends on the cohesive forces and results in higher yield strength and flow dynamics [52–54]. Figure 3b–d, respectively, shows the formation of dimer-, trimer- and pentamer-like structures. Figure 3e shows the 2-D case with the maximum number of capillary bridges. Figure 3f shows a close-up of a capillary bridge between two polystyrene beads with a geometrical representation to evaluate capillary and viscous forces and will be invoked in Sect. 2.4.

**Fig. 4:**
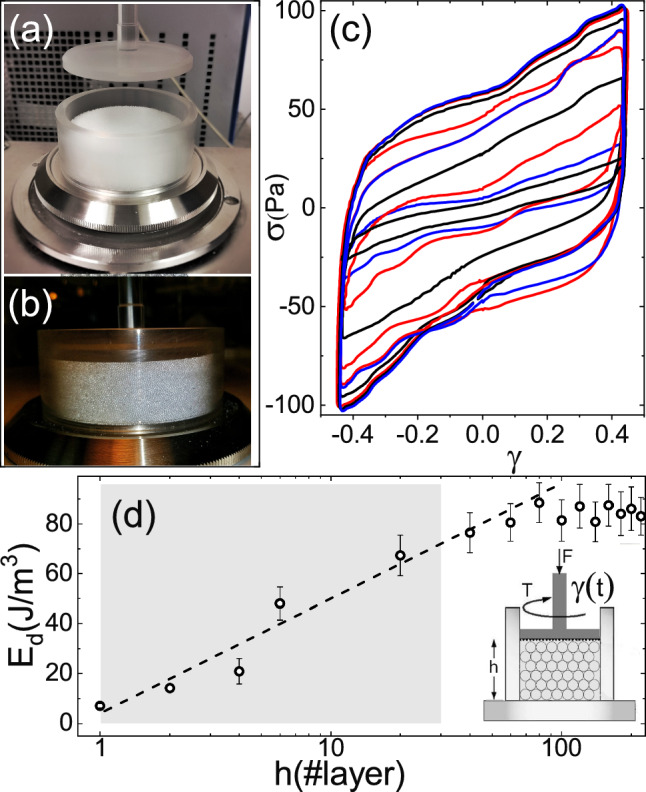
**a** The confined cup–plate geometry as it is in the Haake Mars rheometer set-up and **b** a closer view of the cup with the layers of spherical beads. **c** stress–strain Lissajous loops measured at a frequency of $f={1.5}\,\hbox {Hz}$ and at a strain amplitude of $\gamma _{0}=0.43$ for the increasing number of layers of polystyrene spherical beads of ${500}\,\mu \hbox {m}$ diameter. **d** dependence of the dissipated energy $E_{\text {d}}=\oint \sigma \,d\gamma$ with the number of layers; the grey box indicates the approximate range in which there is no stagnated shear band at the bottom of the cup, and the inset a sketch in which the sample high *h*, the applied normal force *F* and measured torque *T* are indicated

### **Experimental set-up**

Oscillatory rheological experiments on granulates were performed using a Haake Mars II rotational rheometer (Thermo Fisher, Germany) at a room temperature of $T = {23}{}\,^\circ \hbox {C}$. A cup–plate geometry was utilized for shearing the grains (see Fig. 4a, b). We implemented a set-up and protocol similar to that of Lu et al. [21], such that under confinement the gap between the diameter of the plate and the internal diameter of the cup is smaller than the diameter of the grains, with a given initial packing fraction and constant normal force during the measurement. The cup was composed of a Plexiglas cylinder of diameter $d = {50}\,\hbox {mm}$ that permitted seeing the shearing or jamming. The cylinder was firmly attached to the stainless steel bottom plate. The rotating upper plate was made of steel–titanium alloy, which was sandblasted to avoid slipping and to allow uniform stress transmission. Fresh granulates were used for each test. The sample was loaded into the cup and, in the case of dry grains, carefully shaken. For wet grains, the upper plate was rotated for a minute to obtain a homogeneous surface. Both dry and wet grains were subjected to a pre-shearing step at 500 rotations per minute for ${90}\,\hbox {s}$ at the normal force maintained at zero to homogenize the granular assembly. The procedure was performed to achieve an initial height of a predetermined number of layers of equally sized spherical beads by adjusting the initial height until reaching an initial packing of $\phi _{\text {onset}}=0.61$. To repeat the same conditions for all the experimental runs with dry and wet samples, we checked the initial packing fraction after the pre-shearing $\phi _{\text {onset}}$, before and after the rheological measurement $\phi _{\text {offset}}$, by measuring the height of the granular bed.

After the granular sample preparation, the large amplitude sinusoidal shear strain was driven by the plate with a given amplitude $\gamma _{0}$ and frequency *f*. The measured torque was recorded to be represented in stress–strain Lissajous–Bowditch (LB) loops. The experimental runs were conducted in the range $0.005\le \gamma _{0}\le 400$, at which $\gamma _{0}\approx 0.1$ is found consistent with the onset for grain rearrangements and breaking and regeneration of liquid bridges, which also roughly correspond to local displacements of the order of the diameter of the bead; within this range, we identified the range of interest for our study $0.1\lesssim \gamma _{0}\lesssim 7$ as the yielding range.

For a given gap, the rheometer calibration permits evaluation of the stress in the occupied volume of the cup, assuming that the torsional flow within the cup evolves to a steady-state. In the parallel plate geometry and in our particular case of plate and cup, the applied shear strain experienced by the granulates is not uniform, due to which the partial fluidization of the grains prevented all the grains in the cup from contributing to the measured torque. To illustrate this experimentally, Fig. 4c shows several LB-loops for an increasing number of layers from 1 to more than 200, measured in the yielding range at $\gamma _{0}\approx 0.43$. The energy dissipated in one cycle was evaluated for each strain–stress loop by integrating the area of the loop $E_{\text {d}}=\oint \sigma \,d\gamma$. As shown in Fig. 4d, the dependence of $E_{\text {d}}$ on the number of packed layers allowed us to set as high as $h\gtrsim 40$ layers in which the dissipated energy saturates to a constant value corresponding to a stationary shear band. Note that, for the first $h\sim 40$ layers, the dissipated energy increases logarithmically with the number of layers, such that the number of active fluidized beads causing dissipative flow in each layer decreases inversely proportional to the number of layers with offset at the shear plate. Therefore, for experimental practice, we found that approximately 30 layers prevented the formation of a stagnated shear band at the bottom of the cup. For a packing fraction of 0.61, the number of Dynoseeds spheres in the cup can be estimated to be 246593, and assuming that all the beads in the first two layers are fluidized, then the fraction of fluidized beads in the cup resulted in $\sim 0.31$ of the total, equivalent to $\sim 78737$ beads under dissipative flow. Hence, for 30 layers, the measured dissipated energy was ${72}\,\hbox {J}/\hbox {m}^{3}$, and the dissipated energy per active bead should be in the range ${8}\hbox {nJ}/\hbox {bead} \lesssim W_{\text {d}}\lesssim {24}\,\hbox {nJ}/\hbox {bead}$, depending on the fraction of fluidized beads between the total number to the estimated fraction of $\sim 0.31$ of the total.

For all the experiments presented in our study, we reproduced the same initial conditions and the packing fractions were as follows for wet grains $\phi _{\text {onset}}=(0.612 \pm 0.001)$ and $\phi _{\text {offset}}=(0.619 \pm 0.003)$ and for dry grains $\phi _{\text {onset}}=(0.611 \pm 0.002)$ and $\phi _{\text {offset}}=(0.620 \pm 0.005)$. During the experimental runs, the normal force of $F = {1}\,\hbox {N}$ stayed constant, the applied frequency was ${1.5}\,\hbox {Hz}$, and the strain amplitude varied between $\gamma _{0}=0.001$ and 500. For chosen conditions of this experiment, the savage number results ${\text {Sa}}=\rho .(\omega .d/3)^2/(F/(\pi .d^2/2))\sim 0.05$ [21]. We verified that the LB-loops are reproducible and stable during 10 cycles. We checked in the frequency range from ${0.1}\,\hbox {Hz}$ to ${10}\,\hbox {Hz}$ at constant $\gamma _{0}$, for a few selected values, and found that the loss and storage moduli were roughly independent of the frequency. The LAOS test results were previewed in the form of LB-loops on the Rheowin software. In the next step, the raw stress–strain data were exported and subsequently processed with the MITLAOS script (Version 2.1 Beta for MATLAB), to proceed with their orthogonal decomposition and expansion with the Chebyshev polynomials.

### **Liquid bridge rupture energy**

Before we plunge into the report of the experimental results, evaluating the forces between beads and rupture energy of the capillary bridges is necessary, using a schematic approach based on the Derjaguin approximation [55]. The variables and parameters required to estimate the force between particles can be calculated by geometrically tracing a micrographic representation of a typical liquid bridge between two Dynoseeds spheres as shown in Fig. 1f. Thus, the force can be calculated as the sum of capillary and viscous forces as explained by Pitois et al. [56] by Eq. 15:

15 $$\begin{aligned} \begin{aligned} F_{c}&=2 \pi R.\gamma _{\text {oil}}\cdot \cos {\theta }\cdot \zeta _{V}\\ F_{v}&=\frac{3}{2}\pi \cdot R\cdot \gamma _{\text {oil}}\cdot \frac{\mathscr {C}_{a}}{\hat{H}}\cdot \zeta _{V}^2\quad \mathscr {C}_{a}=\frac{\mu \cdot \mathscr {V}_{r}}{\gamma _{oil}}\\ \zeta _{V}&= 1-\frac{1}{\sqrt{1+\frac{2\cdot \hat{V}}{\pi \cdot \hat{H}}}} \end{aligned} \end{aligned}$$

where $\hat{H}=H/R$ is the dimensionless distance between the surface of the two beads of radius *R*, $\hat{V} =\pi \cdot x^2\cdot H/R^3$ is the dimensionless bridge volume, *x* is its azimuthal at its contact line, $\theta$ is the solid–liquid contact angle, $\gamma _{oil}$ is the surface tension of the fluid, $\mu$ is the viscosity of the wetting fluid and $\mathscr {C}_{a}$ the capillary number and $\mathscr {V}_{r}$ is the relative speed of the two beads. For the typical bridges between Dynoseeds polystyrene beads with $R={250}\,\mu \hbox {m}$, as shown in Fig. 1f, we measured $H\approx {48}\,\mu \hbox {m}$, $x\approx {60}\,\mu \hbox {m}$, $\theta \approx {32.3}{}^\circ$, and we got $\hat{H}\approx 0.19$, $\hat{V}\approx 0.035$ and a capillary force $F_{c}\approx {1.5}\,\mu \hbox {N}$. Taking into account the experimental conditions, the possible relative speed between the two Dynoseeds spheres, and the range of deformations in which the rupture and regeneration of the liquid bridges are allowed reveal a possible range for the capillary number as $0.0065\lesssim \mathscr {C}_{a}\lesssim 0.065$. As the oscillatory shear strain in one cycle of period $T=1/f={0.67}\,\hbox {s}$ for $\gamma _{0}\sim 0.1$ corresponds to a relative displacement of the order of the diameter of a bead 2.*R*, relative speed associated with the rearrangement of one bead should be $\mathscr {V}_{r}\sim 2.R/T\sim {0.75}\,\hbox {mm}\,\hbox {s}^{-1}$; whereas for the yielding range, as we report in this article, it appeared as if the possible rearrangements or jumping events from one to ten successive beads jumps in one cycle resulted in a relative speed similar to the viscous force evaluated from Eq. 15 not larger than $F_{v}\sim {24}\hbox {nN}$.

By using the same aforesaid arguments, a typical bridge rupture energy can be estimated from Eq. 16 as shown in [56], as the sum of capillary and viscous rupture energies:

16 $$\begin{aligned} W_{c}= & {} 2 \pi R^2\cdot \gamma _{oil}\cdot \cos {\theta }\cdot A_{1}\nonumber \\ W_{v}= & {} \frac{3}{2}\pi R^2\cdot \gamma _{oil}\cdot \mathscr {C}_{a}\cdot \bigg [{\ln {\frac{A_{2}\cdot \sqrt{\pi }}{(1+A_{2})^2}}-f(\hat{D}_{m}})\bigg ]\nonumber \\ A_{1}= & {} (1+\theta /2)\cdot (1-A_{2})\cdot \hat{V}^{1/3}+\sqrt{\frac{2\cdot \hat{V}}{\pi }}\nonumber \\ A_{2}= & {} \sqrt{1+\frac{2\cdot \hat{V}^{1/3}}{\pi \cdot (1+\theta /2)^2}}\nonumber \\ f(\hat{D}_{m})= & {} \ln {\hat{D}_{m}}-2\cdot \ln {\bigg [\hat{D}_{m}+\sqrt{\hat{D}_{m}^2+\frac{2\cdot \hat{V}}{\pi }}\bigg ]}\nonumber \\{} & {} +\frac{1}{2}\ln {(\pi \cdot \hat{D}_{m}^2+2\cdot \hat{V})} \end{aligned}$$

where $\hat{D}_{m}=D_{m}/R$ is a dimensionless scale for surface asperities and $D_{m}\sim {0.05}\,\mu \hbox {m}$ is an estimated average to account for the surface asperities of the Dynoseeds polystyrene beads. From this evaluation, we obtain a range of possible values for the bridge rupture energy, such as $W_{c}\sim {0.9}\,\hbox {nJ}$ and the viscous contribution not larger than $W_{v}\sim {1.2}\,\hbox {nN}$. For a polystyrene bead with six liquid bridges (coordination 6 as shown in [57]), breaking all the bridges in a jump, the dissipated energy resulted in the range of ${6}\,\hbox {nJ} \lesssim W_{6}\lesssim {12}\,\hbox {nJ}$.

**Fig. 5:**
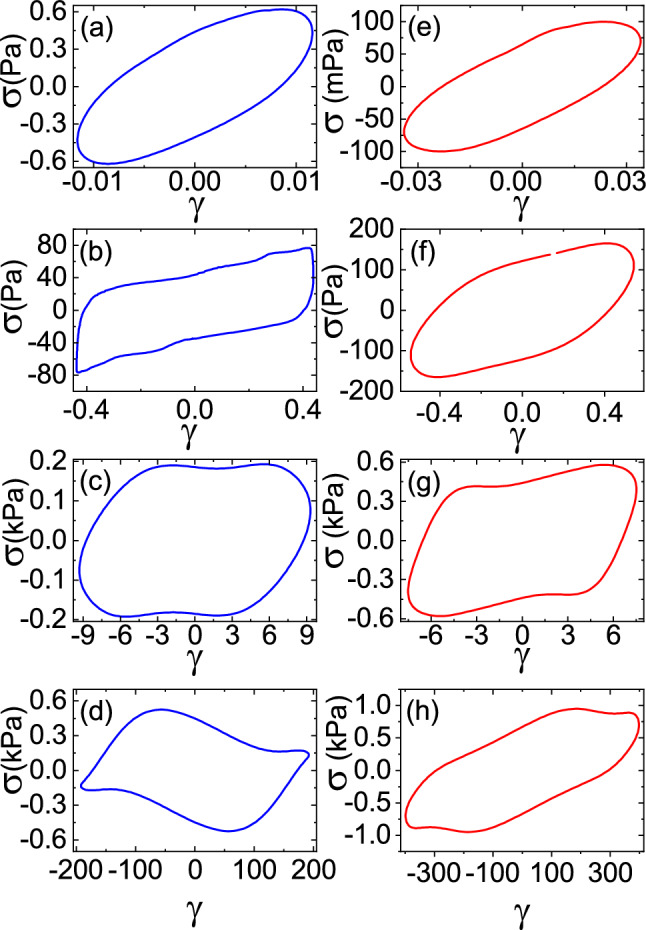
Dynamical regimes of wet (blue) and dry (red) Dynoseeds under large LAOS-strain and under a normal force of $F = {1}\,\hbox {N}$ at a frequency of ${1.5}\,\hbox {Hz}$. Measured typical stress–strain Lissajous loops: in the linear pre-yielding range for wet (**a**) and dry (**e**) granulate; in the nonlinear yielding regime for wet (**b** and **c**) and dry (**f** and **g**) granulates, respectively; and in the slip regime for wet (**d**) and dry (**h**) granulates

## Chebyshev polynomials expansion of the experimental results and discussion

As it was explained above in Sect. 2.3, the experimental results reported in this section were measured by driving confined granular assemblies with a low-frequency controlled sinusoidal strain in time $\gamma (t)=\gamma _{0}\cdot \sin {(\omega t)}$, additionally implying a cosinusoidal strain rate $\dot{\gamma }(t)=\omega \cdot \gamma _{0}\cdot \cos {(\omega t)}$ transmitted by the sensor plate, with a given frequency $\omega = 2\pi f$ and a large strain amplitude $\gamma _{0}$, while the torque and the strain are measured, from which we got the stress response as $\sigma (\gamma _{0},\omega ,t)$. Regarding the dissipated energy per unit of volume in one cycle, we considered our measurements with respect to the beads that were fluidized, expressing its dissipation in units of energy per volume of one bead. In all cases the frequency for this study was selected to be ${1.5}\,\hbox {Hz}$ and the normal force of $F = {1}\,\hbox {N}$ was kept constant. The normal force was enough to maintain the plate in contact with the granulate under confinement without deforming the grains, only following the gentle compaction of the granular assembly during the experiment. Figure 5 shows a few of the stress–strain curves for illustrating different flow regimes for wet and dry Dynoseeds. As we did in our previous work with the ‘tube rheometer’ [27], from these stress strain curves, we were able to identify the pre-yielding strain range and the onset of the yielding range. To discuss here the physical transitions that took place on a time scale shorter than the period of oscillation, we use our previous knowledge from our research on rheological and compaction experiments of granular assemblies, i.e. the oscillatory strain applied through the membranes of the ‘tube rhemometer’ was clearly read in units of the diameter of one grain. The linear viscoelastic behaviour with the characteristic ellipsoid loop is identified in Fig. 5a. In this pre-yielding range, the liquid bridges undergo small elastic deformations until they start to break and regenerate from the onset of the yielding regime at $\gamma _{0}\sim 0.1$, indicated by a non-ellipsoid loop, as shown in Fig. 5b, c. At $\gamma _{0} > 10$, the slipping of grains largely affects the stress–strain response as shown in Fig. 5d with, i.e. $G'_{\text {M}}<0$, characteristic of shear banding [58] possibly due to the coalescence of the liquid bridges locally ending the pendular state and provoking inhomogeneity in the liquid distribution. In the case of dry grains, where the system is governed by frictional forces, in Fig. 5e the stress–strain response is linear, and the dynamics are governed by branching out force chains until the onset for deformations larger than the size of one bead diameter at $\gamma _{0}\sim 0.1$, provoking grain rearrangements and the establishment of new contact points at higher strain until $\gamma _{0} \le 10$. The viscous effects are still more important than elastic ones (Fig. 5f, g). At very large strain, as shown in Fig. 5h, the dry polystyrene beads begin to deform as a solid-like material, causing the stress–strain response to be quasi-Newtonian [59].

**Fig. 6:**
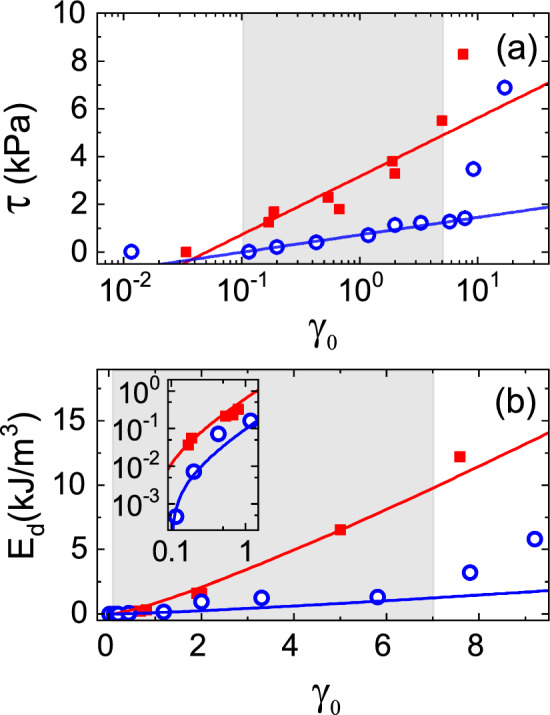
Parameters extracted from the LB-loops of the experimental runs shown in Fig. 5 for dry (red close squares) and wet (blue open circles) Dynoseeds: **a** the loop’s amplitude $\tau$ versus $\gamma _{0}$, and **b** dissipated energy $E_{\text {d}}=\oint \sigma \,d\gamma$ versus $\gamma _{0}$ (where the inset is shown the detail of the smallest strain amplitude range in a log-log graph). In **a** and **b** the lines indicate the corresponding fittings with 17

In relation with our previous work [27], Eq. 2 shows the relation between the storage and dissipated energy measured from the stress–strain LB-loops with a parameter we identified as the noise temperature. In the case study reported here we proceeded in similar way, we evaluated the amplitude $\tau$ and the area $E_{\text {d}}=\oint \sigma \,d\gamma$ of the LB-loops in function of the strain amplitude $\gamma _{0}$ as it is shown in Fig. 6a, b, respectively. The fits according to Eq. 17 are shown that are related to a unique parameter $E_{0}$ identified as the noise temperature. From the data points in Fig. 6a, b, we got $E_{0}=(56.4\pm 0.5)\,\hbox {J}/\hbox {m}^{3}$ and $E_{0}=(16.7\pm 0.5)\,\hbox {J}/\hbox {m}^{3}$ for the dry and wet granulate, respectively, for the range $0.1\lesssim \gamma _{0}\lesssim 7$. As we explained in the introduction the parameter $E_{0}$ is a kind of characteristic energy density and by considering the volume of one bead and the fraction of the fluidized beads in the cup, estimated in Sect. 2.3, we got in terms of energy per active bead: $\sim {19}\,\hbox {nJ}$ and $\sim {6}\,\hbox {nJ}$ for the dry and wet granulate, respectively; thus, we found that we can get into similar discussion as we did related with our experiments with the ‘tube rheometer’; from this analysis of our findings, in the next sections we will examine it from the point of view of the Chebyshev coefficients.17

### **First harmonic elastic and viscous moduli**

**Fig. 7:**
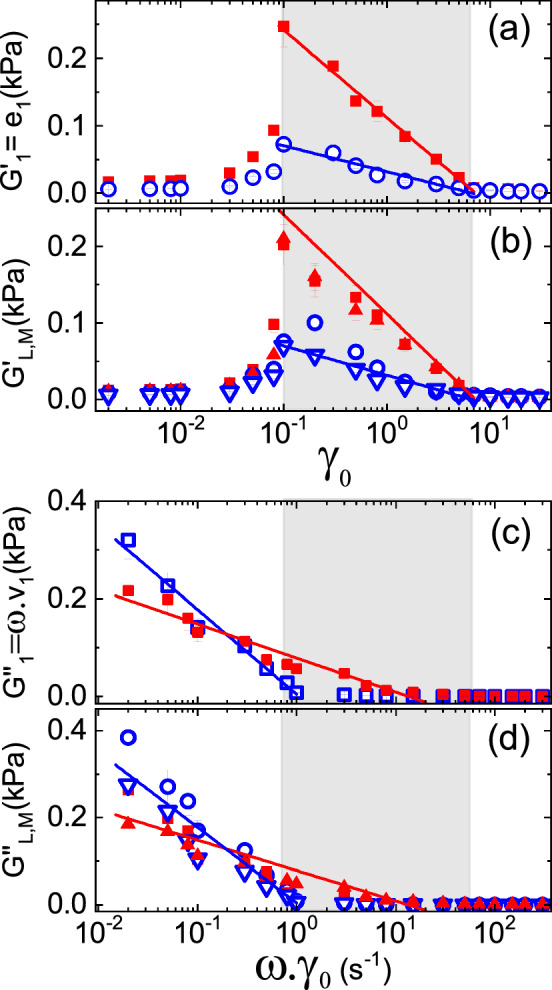
Semi-logarithmic representations of the first harmonic elastic (**a**) and viscous (**c**) moduli $\textrm{G}'_{1}$ and $\textrm{G}''_{1}$, or first Chebyshev coefficients $e_1$ and $v_1$ as a function of strain amplitude and the strain rate amplitude $\omega \gamma _0$, for dry (closed symbols) and wet (open symbols) Dynoseeds beads, respectively, where $\omega = {9.4}\,\hbox {rad}\,\,\hbox {s}^{-1}$. Corresponding fits in (**a**) and (**c**) were evaluated with Eqs. 18 and 19. Semi-logarithmic representations of (**b**) the nonlinear elastic moduli as a function of strain amplitude $\gamma _0$, where squares and circles: secant elastic modulus $\textrm{G}'_\textrm{L}$, triangles: tangent elastic modulus $\textrm{G}'_\textrm{M}$; and the (**d**) nonlinear loss moduli as a function of the strain rate amplitude $\omega \gamma _0$, where squares and circles: secant loss modulus $\textrm{G}''_\textrm{L}$, triangles: tangent loss modulus $\textrm{G}''_\textrm{M}$. The corresponding fits of (**a**) and (**c**) were used in (**b**) and (**d**) as a reference to show the agreement in the slope attributed to the ‘noise temperature’ (for detailed explanation see text). The grey box indicates the range $\gamma _{0}^\text {onset} \le \gamma _{0} \le \gamma _{0}^\text {offset}\text {,}$ defined from (**a**) by the fits with Eq. 18

Using the MITLAOS framework applied to the measured stress–strain loops by its orthogonal decomposition and fitting the Chebyshev expansion to their symmetry lines, we evaluated its coefficients. In parallel, we extracted the tangent and secant moduli by applying the approach as geometrically shown in Fig. 2. Then as shown in Fig. 7a, c, we were able to obtain the first harmonic elastic and viscous moduli $G'_{1}$ and $G''_{1}$ as functions of the strain amplitude $\gamma _{0}$ and the evaluated strain rate amplitude $\omega \gamma _{0}$, respectively. Similarly, the tangent and secant moduli are shown in Fig. 7b, d. At small deformations $\gamma _{0}\lesssim 0.05$, we observed all harmonics of the viscoelastic moduli showed practically no dependence on the strain amplitude in the pre-yielding regime. The yielding onset $\gamma _{0}^{\text {onset}}\approx 0.1$ indicates the regime change at the maximum of the moduli. These moduli decrease significantly faster for dry grains than for wet grains, suggesting the rearrangement of the mesoscopic-scale structures caused by shear fields. In this deformation range, grains are under the influence of anisotropic forces, which are eventually dissipated by non-affine motions, though at varying relaxation time scales [59]. In the case of the wet granulate, the rearrangement of the liquid bridge network is dissipated by releasing the capillary energy. At high strain, the liquid between adjacent grains would squeeze out due to compressive stresses and thus the pendular state of the liquid bridge network would be destroyed; in both cases, for high deformations, strain amplitude $\gamma _{0}>7$ in the slip regime was developing shear banding and got jammed and eventually rotated as a single body.

The Chebyshev coefficients should be understood as viscoelastic moduli. We shall discuss them in our particular case of powders, as we did in our previous work of the reference [27] with the proposed Eq. 2, and exploring them through a semi-logarithmic representation of the elastic and viscous first harmonic, as shown in Fig. 7a, c. We proposed to fit the first elastic harmonic in the nonlinear range with Eq. 18:18From the data points in Fig. 7a, we got the following fitting parameters: $\gamma _{0}^{\text {offset}}=(7.3 \pm 0.6)$, $G_{1}^{'0}=(56 \pm 1)\, \hbox {J}/\hbox {m}^{3},$, and $\gamma _{0}^{\text {offset}}=(7 \pm 2),$, $G_{1}^{'0}=(17\pm 1)\, \hbox {J}/\hbox {m}^{3}$ for the dry and wet granulate, respectively. These results were found consistent with what we were discussing in the previous section related to Fig. 6. The characteristic modulus is assumed to be proportional to a kind of characteristic energy that set the state of the system dynamics, $G_{1}^{'0}\cdot \nu \propto E_{1}^{'0}$; as we defined in [27] with $\nu$ the volume of one bead and considering only the fraction of fluidized beads in the cup, estimated in Sect. 2.3, we got $E_{1}^{'0}\sim {19}\,\hbox {nJ}$ and $E_{1}^{'0}\sim {6}\,\hbox {nJ}$ for the dry and wet granulate, respectively. Given that $\gamma _{0}^{\text{ onset }}=0.1$, we found $\gamma _{0}^{\text {offset}}\sim 7$ corresponds to the onset of the wall-slip regime as previously discussed. Following up on our previous work as shown in [27], in the yielding range, it seems appropriate to understand this characteristic energy for the wet granulates, as the noise temperature governing the dynamics of breaking and regeneration (br) of liquid bridges $E_{1,{\text {br}}}^{'0}\equiv k_{B}\cdot \Theta _{{\text {br}}}\sim {6}\,\hbox {nJ}$, or $\Theta _{{\text {br}}}\sim {0.4}\,\hbox {PK}$ (peta Kelvin). In Sect. 2.4 the bridge rupture energy range was estimated to be ${1}\,\hbox {nJ} \lesssim W\lesssim {2}\,\hbox {nJ}$, assuming a coordination six, being of the order of the noise energy set by $k_{B}\cdot \Theta _{\text {br}}$. In the case of the yielding range for the dry granulate, the noise temperature governing the dynamics of grain rearrangement (gr) should be $E_{1,{\text {gr}}}^{'0}\equiv k_{B}\cdot \Theta _{{\text {gr}}}\sim {19}\,\hbox {nJ}$ or $\Theta _{\text {gr}}\sim {1.4}\,\hbox {PK}$. Besides also in this range, the smaller noise temperature for the wet granulate than for the dry $\Theta _{\text {br}} < \Theta _{\text {gr}}$ corroborates the finding that, under confinement, the wet granular assembly flows dissipating less energy than the energy dissipated by the dry one [27].

We also conducted a similar analysis for the viscous first harmonic, with Eq. 19 written for different ranges identified in Fig. 6b, for the wet $\gamma _{0} \le \gamma _{0}^\text {onset}$ and dry powder $\gamma _{0} \le \gamma _{0}^\text {offset}$ as follows:19From the fittings, we obtained the following for the wet powder in the pre-yielding range: $\gamma _{0}^{\text {onset}}=(0.11 \pm 0.02)$ and $G_{1}^{''0}=(8.0 \pm 0.4)\, \hbox {J}/\hbox {m}^{3}$ and for the dry powder in the range $\gamma _{0}\lesssim 2$, we got: $\gamma _{0}^{\text {offset}}=(1.5 \pm 0.5)$ and $G_{1}^{''0}=(3.2 \pm 0.3)\, \hbox {J}/\hbox {m}^{3}$. Similar to the evaluations made for the elastic first harmonic, we thus rewrite these results considering the number of fluidized beads and changing units following our assumption that the characteristic loss modulus is also proportional to a characteristic viscous energy $G_{1}^{''0}\cdot \nu \propto E_{1}^{''0}$, for the wet granulate: $E_{1}^{''0}\sim {3}\,\hbox {nJ}$ for the pre-yielding range and for the dry beads: $E_{1}^{''0}\sim {1}\,\hbox {nJ}$ for $\gamma _{0}\lesssim 2$. Furthermore, in the pre-yielding range, the viscous first harmonic for the wet granulate revealed a major role compared to the elastic one $\gamma _{0} \lesssim \gamma _{0}^\text {onset}$ (Fig. 7b); in this range the shear strain oscillations are not provoking displacements enough to break up liquid bridges; thus, this viscous dissipation should be related to the oscillations in the liquid bridge network. From the onset of breaking and regeneration dynamics, this viscous dissipation mode is interrupted almost to zero. In the case of the dry granulate, we observed a range for $\gamma _{0} < \gamma _{0}^\text {offset}$, where the viscous dissipation should be driven by friction between grains either in the pre-yielding range but also from the onset of particle rearrangements, where the offset of this viscous friction range could correspond to the offset for the force chains branching out. It should be also valid to extend the concept of noise temperature since we are describing a different ensemble of inherent states [26]; thus, we could write for the noise temperature governing the viscous oscillations of the liquid bridge network in the pre-yielding range $E_{1,{\text {lbn}}}^{''0}\equiv k_{B}\cdot \Theta _{\text {lbn}}\sim {3}\,\hbox {nJ}$ or $\Theta _{\text {lbn}}\sim {0.2}\,\hbox {PK}$; and for the noise temperature governing the viscous friction of the grain contacts (*f*) $E_{1,f}^{''0}\equiv k_{B}\cdot \Theta _{f}\sim {1}\,\hbox {nJ}$ or $\Theta _{f}\sim {0.07}\,\hbox {PK}$.

**Fig. 8:**
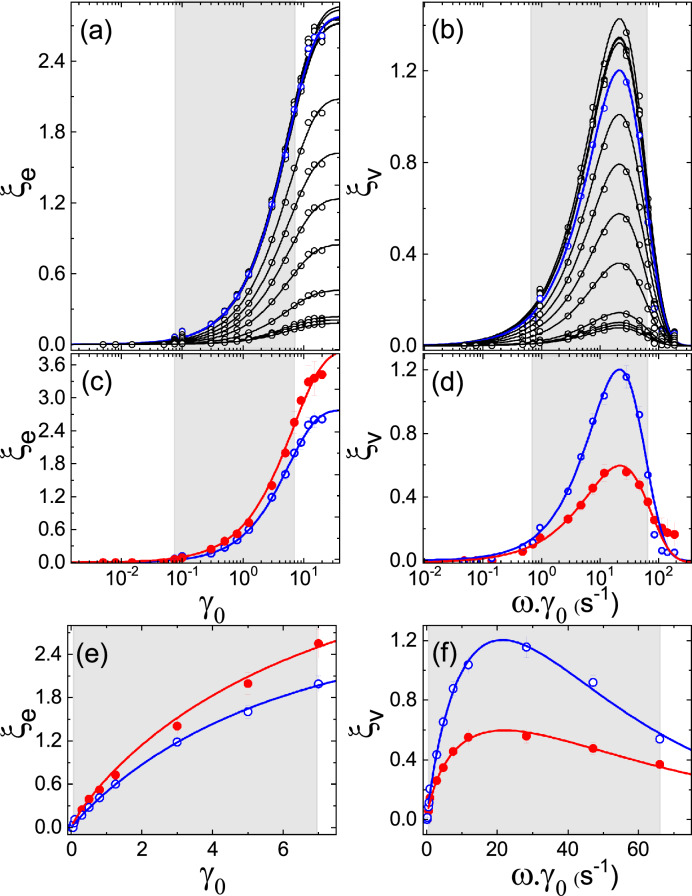
Semi-logarithmic representation of the amount of nonlinearities as a percentage of the first Chebyshev coefficients, evaluated with Eq. 21, from the experimental data for polystyrene beads of ${500}\,\mu \hbox {m}$ with and without the small addition of silicon oil. For the wet granulate: **a** elastic nonlinearity $\xi _{e}$ as a function of the strain amplitude $\gamma _{0}$ and **b** viscous nonlinearity $\xi _{v}$ as a function of the strain rate quantified as $\omega \gamma _{0}$, the measurements were done at different oscillation frequencies, starting from the bottom for the curves are ordered as follows: 0.01, 0.05, 0.1, 0.3, 0.5, 0.75, 1, 1.25, 1.5, 2, 3, 5 and ${10}\,\hbox {Hz}$. The corresponding fittings for $\xi _{e}$ and $\xi _{v}$ with Eq. 23 of the peak curve and adjusted together with Eq. 22 (for detailed explanation, see text). The curve depicted in blue corresponds to the frequency selected for our study at ${1.5}\,\hbox {Hz}$ to investigate the nonlinearities for the wet and dry granular assembly; data represented as open and close symbols, with the corresponding fittings in blue and red, respectively; as is shown in **c** and **d** their semi-logarithmic and in **e** and **f** their linear representations. The grey box indicates the fittings range

### **Higher-order viscoelastic moduli contributions to nonlinearities**

Taking into account Eqs. 11 to 14 makes easier to identify what is shown in Fig. 7c, d, using the corresponding fits of (a) and (b) with Eqs. 18 and 19 in (c) and (d) as a reference, we observed the agreement in the slope and the small deviations from it, which correspond to the contribution of higher harmonics. We should note that the intensities of the higher harmonics decreases considerably being the third, fifth and seventh harmonic of the order of $1\%$, $0.1\%$ and $0.01\%$ of the first harmonic. We also explore the oscillation frequencies in the range from ${0.01}\,\hbox {Hz}$ to ${10}\,\hbox {Hz}$ noticing a dependence on frequency only for higher harmonics. Rewriting Eqs. 11 to 14 helps us rule out the first harmonic and collapse the modulus to reveal pure nonlinear response, as shown in Eq. 20 for the third harmonic:

20 $$\begin{aligned} \begin{aligned} e_3 \sim \frac{(G_{\text {M}}^{'}-G_{\text {L}}^{'})}{3} \,\,\,\,\,\,\, v_3 \sim \frac{(\eta _{\text {L}}-\eta _{\text {M}})}{3} \end{aligned} \end{aligned}$$

Analogous to the Fourier transform-derived Q-parameter (ratio of the relative intensities) for quantifying nonlinearity present in complex materials [39], we scaled Chebyshev polynomial of third, fifth, and seventh to the first kind to estimate the possible amount of nonlinearity present in the system. Equations 21 were used to evaluate the elastic $\xi _\text {e}$ and viscous $\xi _\text {v}$ nonlinearities expressed as percentage of the first Chebyshev coefficients.

21 $$\begin{aligned} \begin{aligned} \xi _{e}&= 100\cdot \sqrt{\frac{e _ {3}^2 + e _ {5}^2 + e _ {7}^2}{e _ {1}^2}}\\ \xi _{v}&= 100\cdot \sqrt{\frac{v _ {3}^2 + v _ {5}^2 + v _ {7}^2}{v _ {1}^2}} \end{aligned} \end{aligned}$$

These nonlinearities are shown in Fig. 8; in the pre-yielding regime, $\xi _\text {e}\sim \xi _\text {v}\approx 0$, the behaviour is showing only a small amount of nonlinearity for the strain amplitude increasing in the vicinity to the yielding onset, and it is consistent with nonlinear viscoelastic moduli as shown in Fig. 7. Positive values of $\xi _\text {e}$ indicate strain stiffening among dry and wet granules. $\xi _\text {e}^\text {dry} > \xi _\text {e}^\text {wet}$ for a given strain amplitude. This implies that dry grains are stiffer than wet grains, which agrees with previous studies on the rheology of dense granular pastes [27, 51, 60]. In the yielding regime, as it happens from $\gamma _{0}^{\text {onset}}\approx 0.1$ as observed in Fig. 8a, $\xi _\text {e}$ gradually increased until saturation while $\xi _\text {v}$ gave a peak behaviour, indicating a possible relation between elastic and viscous nonlinearities $\xi _\text {e}$ and $\xi _\text {v}$. Under small deformation $\gamma _{0}<0.05$, the granular assembly is governed by linear elasticity and remains largely unaffected, instigating the networks of liquid bridges and force chain branches. Under large deformation, in the yielding regime, rearrangement processes lead to a finite number of nonlinear events. In order to investigate this nonlinear elastic response of the granulates, we propose to fit the elastic nonlinearity with the stretched exponential Eq. 22:

22 $$\begin{aligned} y= \dfrac{\xi _{e}}{\xi _{e}^\infty }= 1-\exp -x^\delta , \,\ \,\ \,\ \,\, x= \dfrac{\gamma _{0}^{}}{\gamma _{0}^\infty } \end{aligned}$$

To analyse the viscous nonlinearity, we consider the relation between steady-state rheology and compaction experiments. In both studies, the origin of the deviation from linearity is related to the rearrangements in the granular assembly, which is identified as nonlinear events. Experimental studies on granular compaction by Lumay and Vandewalle [16] examined the dynamics at three different spatial and temporal scales: (1) evolution of the packing fraction to its saturation value fitted by the Kohlrausch–Williams–Watts law $\tilde{\rho }=1-\exp -(t/\tau )^\beta$, where $\tau$ is the relaxation time and $\beta$ is the stretched exponent; (2) evolution of mesoscopic domains by correlating the mesoscopic packing fraction $\tilde{\phi }$ with the macroscopic packing fraction $\tilde{\rho }$ through a power function, stated as $\tilde{\phi }=\tilde{\rho }^2$, which, in the mentioned study [16] is the determining the diffusion-controlled growth of mesoscopic domains for granulates in two dimensions; and (3) that at the microscopic scale, the mobility of grains is proportional to the variation of the packing fraction induced by an external force, $\mu \sim {\text {d}}\tilde{\rho }/{\text {d}}t$.

The evolution of the elastic nonlinearity $\xi _\text {e}$ until saturation, as shown in Fig. 8, is governed by the growth and coalescence of mesoscopic domains and, therefore, should be scalable with the macroscopic packing fraction $\tilde{\rho }$. Being granular matter rheology under the same dynamics as in a compaction experiment, the source of viscous nonlinearity $\xi _\text {v}$ can be corroborated with the rearrangement of mesoscopic domains induced by external shear forces. Following Lumay and Vandewalle [16] and assuming the proportionality $\xi _\text {v}\sim {\text {d}}\tilde{\phi }/{\text {d}}t$, we propose Eq. 23 to investigate the relation between elastic and viscous nonlinearity.

23 $$\begin{aligned} \dfrac{\xi _{\nu }}{\xi _{v}^\infty }\sim \Bigl [\dfrac{{\text {d}}y^2}{{\text {d}}x}\Bigr ]_{k.x},\quad k.x=\dfrac{\omega \gamma _{0}}{(\omega /k)\gamma _{0}^\infty } \end{aligned}$$

where *k* is a parameter to adjust the position of data curves on the axis with $\omega \gamma _{0}$ so the extended exponent $\delta$ and $\gamma _{0}^\infty$ are kept the same for both Eqs. 22 and 23. We tested the consistency of the proposed relation between the elastic and viscous nonlinearities by fitting the evaluated nonlinearities, processing the data from oscillatory strain experiments for different frequencies in the range from ${0.01}\,{Hz}$ to ${10}\,{Hz}$ on polystyrene beads with a small quantity of silicon oil, enough to reach the pendular state for the liquid bridge network as shown in Fig. 8a, b. By fitting the data shown in Fig. 8a, b, we obtain for all the curves in the applied range of frequencies: $k=(1.581 \pm 0.003)$, $\delta =(0.949 \pm 0.001)$ and $\gamma _{0}^\infty =(5.62 \pm 0.01)$. As already stated above in Sect. 2.3 we chose the frequency ${1.5}\,\hbox {Hz}$ for our experiments to study dry and wet granulates, as the corresponding fits are shown in blue colour in Fig. 8a, b. Figure 8c, d depicts the semi-logarithmic representation of the elastic and viscous nonlinearities for the dry and wet granulate, and similarly, the respective linear representation is shown in Fig. 8e, f, to support the agreement with the fittings. For the dry powder, as shown in red, fitting the data revealed: $k=(1.55 \pm 0.02)$, $\delta =(0.870 \pm 0.005)$, and $\gamma _{0}^\infty =(6.69 \pm 0.05)$. We identified $\gamma _{0}^\infty$ as a measure of the elastic range of the granulate where the elastic range for dry granulate is found larger than the granulate with small additions of silicon oil. In contrast to the elastic nonlinearities $\xi _\text {e}^\text {dry} > \xi _\text {e}^\text {wet}$, the peak functions for the viscous nonlinearities $\xi _\text {v}^\text {dry} < \xi _\text {v}^\text {wet}$ allude to the inherent states or the noise dynamics at the mesoscopic scale, wherein the viscous nonlinearity is associated with an enhanced flowability in the mesoscopic scale due to a larger variation in the mesoscopic packing fraction, as it was also indicated in the yielding range by their noise temperatures $\Theta _{\text {br}} < \Theta _{\text {gr}}$.

## Conclusions and outlook

We investigated dry and wet granular matter flow by running low-frequency oscillatory strain sweeps experiments with a rotational rheometer using a cup-and-plate geometry, applying large deformation under constant normal pressure, enough to maintain the contact of the plate with the granular assembly. To evaluate the results of these experiments on cohesive powders, we developed a methodology based on the concept of noise temperature as introduced by Sollich [22] within the soft glassy materials model (SGM) but also validated as a genuine thermodynamic configurational temperature as it is in the frame of the shear transformation zone theory (STZ) [25, 26]. The orthogonal decomposition of stress developed by Cho et.al [35] and extended by Ewoldt et al. [36] permitted us to quantify our rheological data into elastic and viscous stress Lissajous–Bowditch (LB) loops. From these loops, it was possible to approximate their symmetry lines with Chebyshev polynomials of the first kind. The strain sweeps experiments permitted us to observe by increasing the strain amplitude, the pre-yielding and yielding regimes followed by a slip-stick regime. The onset of yielding $\gamma _{0}^\text {onset}=0.1$ was identified by the abrupt increase in the storage and loss moduli with respect to the applied strain amplitude, which agreed with our previous investigation [27]. In the yielding range $\gamma _{0}^\text {onset}\lesssim \gamma _{0}\lesssim \gamma _{0}^\text {offset}$, nonlinear events are driven by rearrangements of the dry and wet granular assembly, which was confirmed from the analysis of elastic and viscous nonlinearities, evaluated from the high-order Chebyshev coefficients. The offset of the yielding regime $\gamma _{0}^\text {offset}$ is indeed the onset of the slip-stick regime, which in the case of the wet granulate corresponded to the end of the liquid bridge’s pendular state provoking the coalescence of the liquid bridges and creating shear banding [58], while in the case of the dry granulate, it got jammed due to very large deformation spinning as a single body of agglomerated polystyrene beads. Elastic and viscous Chebyshev coefficients related to the Fourier decomposition are the nonlinear storage and loss moduli of the granular assembly; we attempted to understand them in the pre-yielding and yielding regime in relation to the noise-driven dynamics of the ensemble of mesoscopic elements that determined the flow behaviour in each regime.

The SGM model [23, 24] describes the energy landscape of soft glassy materials as an ensemble of mesoscopic elements, each storing elastic energy, in which the jumping of these elastic elements over strain-modulated energy barriers is activated by a non-thermal temperature. On this premise and a model based on granular compressibility, by retaining the Coulomb yield conditions and dilatancy behaviour, Lu et.al [21] have shown experimentally that the steady-state rheology and the compaction behaviour of powders are related as a part of the theory of jamming [20]. Contributing to this research line, in previous work, we carried out compaction experiments [28, 29] in which we tested an energetic approach developed by Ludewig et.al [61]; a kinetic equation in terms of an energy parameter as the sum of the kinetic and potential energy for each tap described compaction dynamics having an Arrhenius-like exponential factor with the dimensionless ratio between a characteristic energy of the barrier and the injected energy in each tap as its argument. Thus, a clear dynamics of injection of energy in each tap followed by the jumping of energy barriers between local energy states has been experimentally validated [28, 29]. Moreover, evaluating stress–strain LB-loops revealed a scaling relation between the strain amplitude $\gamma _{0}$ and the storage elastic energy, which was made possible by a ‘tube rheometer’ applying oscillatory strain in a single shear band granular assembly [27]. In the yielding range $\gamma _{0}^\text {onset}\lesssim \gamma _{0}\lesssim \gamma _{0}^\text {offset}$, the amplitude $\gamma _{0}$ of the strain oscillation was shown to be proportional to a Boltzmann factor with its argument containing what was identified as the noise temperature, also named the configurational or disorder temperature. This observation is also consistent with the basic idea of the STZ theory in which the population density of shear transformation zones *n* should be proportional to a Boltzmann factor in which the disorder temperature is in its argument [26]. In our case, the density of the activated mesoscopic elements should be proportional to the strain amplitude $n\propto \gamma _{0}$.

The expansion with Chebyshev polynomials of the symmetry lines of the experimental strain–stress LB-loops permitted us to investigate the dependence of the viscoelastic Chebyshev coefficients on the strain amplitude. It was found for the first harmonics, elastic and viscous, the proportionality of the amplitude of the strain oscillation with a Boltzmann factor, where its argument resulted to be the dimensionless ratio between the energy density of the viscoelastic elements and the noise temperature governing the dynamics of these mesoscopic elements. Note that this scaling is universal and is also valid for a wide variety of glasses as the experimental evidence was recently reported by Song et al. [62]. In their stress relaxation experiments with metallic glasses, the STZ as dynamic variable describes the flow of local atomic configurations induced by shear strain; our experiments on the evolution of mesoscopic grain configurations induced by deformation [27] agree with the reported behaviour.

Then we would have to emphasize the central role of configurational entropy in the dynamics of granular matter [25, 26, 63, 64]. Commonly used in powder engineering to numerically simulate manufacturing facilities, the granular temperature is understood as a conventional kinetic temperature, while the configurational or noise temperature is a genuine thermodynamic temperature that satisfies the statistical meaning of temperature as $1/\Theta \equiv \partial S/\partial U$, in which *U* is the internal energy as pointed out first by Hong and Hayakawa [63]. The development of this non-equilibrium thermodynamic view has the potential to contribute not only to a better understanding of granular matter dynamics but also to be applied to breakthrough innovations in powder technologies [65, 66]. In our previous work by considering the potential gravitational energy, for the evaporation transition of granular matter, a configurational temperature of $\Theta \sim {9}\,\hbox {PK}$ (peta Kelvin) was experimentally found [64]. This order of magnitude in temperature is difficult to grasp, being peta Kelvins only theoretically estimated for the quark epoch of the big-bang theory timeline [67]; however, it made sense corresponding to inherent states described as slow configurational degrees of freedom that maximize the configurational entropy. For the pre-yielding and yielding regime, from the expansion of the experimental LB-loops with Chebyshev polynomials, it was possible to evaluate the corresponding noise temperature. In the pre-yielding regime, the network of grain contacts of the dry granulate were the source of the viscous dissipation. Its dynamics were set by a noise temperature $\Theta _{f}\sim {0.07}\,\hbox {PK}$, while for the granulate with a silicon oil bridge network, in a pendular state, the temperature setting their oscillation dynamics was found to be $\Theta _{\text {lbn}}\sim {0.2}\,\hbox {PK}$. In the yielding range, a noise temperature of $\Theta _{\text {gr}}\sim {1.4}\,\hbox {PK}$ set the noise-driven dynamics of grain rearrangements, while a noise temperature of $\Theta _{\text {br}}={0.4}\,\hbox {PK}$ set the dynamics of breaking and regeneration of liquid bridges, being also consistent with our estimation for the liquid bridge rupture energy. This noise temperature scale agrees with our experiments with the ‘tube rheometer’, in which we measured for dry sand with increasing packing fraction, a range of ${1.5}\,\hbox {PK}\lesssim \Theta _{\text {gr}}\lesssim {6.4}\,\hbox {PK}$, while for the sand with small additions of water, it was found to be $\Theta _{\text {br}}\sim {1.5}\,\hbox {PK}$ [27].

The higher-order Chebyshev coefficients were evaluated to quantify the degree of elastic and viscous nonlinearity $\xi _{\text {e}}$ and $\xi _{\text {v}}$, respectively. For dry grains, the elastic nonlinearity $\xi _{\text {e}}$ was found to be higher than for wet grains. We found the elastic nonlinearity in the yielding range related to a jamming density, also related with the macroscopic compaction of the granular assembly. This finding is consistent with our observation that $\xi _\text {e}^\text {dry} > \xi _\text {e}^\text {wet}$; while in contrast, we observed for the peak function of the viscous nonlinearity that $\xi _\text {v}^\text {wet} > \xi _\text {v}^\text {dry}$. What we found is related with flowability and consistent with the configurational entropy of the granular media as their states are characterized by noise temperatures governing different dynamics of grain rearrangements (gr) and breaking and regeneration of liquid bridges (br), $\Theta _{\text {br}} < \Theta _{\text {gr}}$. We also identified the elastic range of the granular material as $\gamma _{0}^\infty$, a measure of the strain amplitude necessary for the elastic nonlinearity to saturate and also a measure of the jamming point, which from what we found is shorter for the wet granulate than for the dry, in which as it is known, friction extends the elastic range of granular matter [68].

We found a relationship between the elastic and viscous nonlinearity by assuming a relation between the elastic nonlinearity and the macroscopic packing fraction and between the viscous nonlinearity and the derivative of the mesoscopic packing fraction. With respect to the strain amplitude, from the evaluated nonlinearities, the proportionality of the viscous nonlinearity and the variation of the square of the elastic nonlinearity induced by deformation were fitted. This relation further signifies a correspondence between the dynamics of structures such as rattlers in the mesoscopic scale with the macroscopic response of the granular media, in agreement with the discussion by Kumar and Luding [69] associating the origin of nonlinearity to density fluctuations moving the jamming point. As shown by Shi et al. [70], although it is possible to simulate granular matter dynamics with a linear force model, the result showed a nonlinear response originating from the combination of local characteristic time scales associated with the different interactions between grains and a common global time scale related to the grain rearrangement phenomena induced by the confinement pressure. This also points out that the existence of the proportionality between viscous nonlinearity and the variation in elastic nonlinearity means an entropic origin of the nonlinear dynamics of granular matter.

## Data Availability

The measurements and datasets analysed in the current study as shown in the figures of this manuscript are available as ASCII files from the corresponding author on reasonable request.
